# Revolutionizing Clinical Microbiology Laboratory Organization in Hospitals with *In Situ* Point-of-Care

**DOI:** 10.1371/journal.pone.0022403

**Published:** 2011-07-19

**Authors:** Stéphan Cohen-Bacrie, Laetitia Ninove, Antoine Nougairède, Rémi Charrel, Hervé Richet, Philippe Minodier, Sékéné Badiaga, Guilhem Noël, Bernard La Scola, Xavier de Lamballerie, Michel Drancourt, Didier Raoult

**Affiliations:** 1 Fédération de Microbiologie Clinique, Assistance Publique des Hôpitaux de Marseille-Pôle des Maladies Infectieuses, Hôpital la Timone, Marseille, France; 2 Unité de Recherche sur les Maladies Infectieuses et Tropicales Emergentes, CNRS-UMR 6236, IRD 198, IFR 48, Faculté de Médecine, Université de la Méditerranée, Marseille, France; 3 Unité des Virus Emergents, UMR190, IRD and Université de la Méditerranée, Marseille, France; 4 Service d'Accueil des Urgences, Assistance Publique des Hôpitaux de Marseille, Hôpital Nord, Marseille, France; 5 Observatoire Régional des Urgences Provence-Alpes-Côte d'Azur, Hyères, France; Charité, Campus Benjamin Franklin, Germany

## Abstract

**Background:**

Clinical microbiology may direct decisions regarding hospitalization, isolation and anti-infective therapy, but it is not effective at the time of early care. Point-of-care (POC) tests have been developed for this purpose.

**Methods and Findings:**

One pilot POC-lab was located close to the core laboratory and emergency ward to test the proof of concept. A second POC-lab was located inside the emergency ward of a distant hospital without a microbiology laboratory. Twenty-three molecular and immuno-detection tests, which were technically undemanding, were progressively implemented, with results obtained in less than four hours. From 2008 to 2010, 51,179 tests yielded 6,244 diagnoses. The second POC-lab detected contagious pathogens in 982 patients who benefited from targeted isolation measures, including those undertaken during the influenza outbreak. POC tests prevented unnecessary treatment of patients with non-streptococcal tonsillitis (n = 1,844) and pregnant women negative for *Streptococcus agalactiae* carriage (n = 763). The cerebrospinal fluid culture remained sterile in 50% of the 49 patients with bacterial meningitis, therefore antibiotic treatment was guided by the molecular tests performed in the POC-labs. With regard to enterovirus meningitis, the mean length-of-stay of infected patients over 15 years old significantly decreased from 2008 to 2010 compared with 2005 when the POC was not in place (1.43±1.09 versus 2.91±2.31 days; p = 0.0009). Altogether, patients who received POC tests were immediately discharged nearly thrice as often as patients who underwent a conventional diagnostic procedure.

**Conclusions:**

The on-site POC-lab met physicians' needs and influenced the management of 8% of the patients that presented to emergency wards. This strategy might represent a major evolution of decision-making regarding the management of infectious diseases and patient care.

## Introduction

Health care policies are heterogeneous worldwide, but a global effort is striving to achieve a higher value of care delivery. Current strategies are designed to combine quality of care and cost containment [1], [2]. With regard to medical biology, facilities were generally reorganized into core laboratories in the United States beginning in the 1990s [3], [4], and this was supported by the automation of tests [5], [6]. European laboratories are currently following the trend toward centralization of biomedical analyses [7]. Although this organization is cost-effective, the distance from the laboratories to the site of patient care and the batch processing of clinical specimens make rapid turn-around times impossible [3], [6]. Consequently, microbiology laboratories are unable to contribute to timely decision-making for most infectious diseases [6]-[8], resulting in unnecessary treatment and hospitalization [9] as well as the empiric use of antibiotics.

To resolve the time lag between test results and patient care, a growing range of rapid diagnostic tests that can be performed at the point of care (POC) has been implemented [10], [11]. Although most POC tests rely on immuno-chromatographic or agglutination assays, miniaturization and full automation of molecular methods allow for quicker real-time PCR-based detection of pathogens using simplified procedures [10], [12]. These tests address the need to hospitalize patients, to isolate contagious individuals and to initiate and focus anti-infective therapy. For example, the rapid testing of Group B *Streptococcus* colonization in pregnant women at delivery enables timely, focused prophylaxis of materno-fetal infections [13], [14].

In Marseilles, a core laboratory facility serving all five university hospitals (3,500 beds) was adopted for economic purposes. Delays of test results inherently increased as a consequence of the batch processing of higher demand volumes and the geographical distance from hospitals to the laboratory. Thus, a strategy was attempted that used a laboratory fully dedicated to microbiology POC testing. This pilot “POC-lab”, the la Timone POC-lab, was located close to both a pediatric emergency department and our core laboratory to evaluate feasibility. In May of 2008, a second POC-lab was then implemented inside the emergency department of the most distant hospital, Hôpital Nord, which is an 800-bed healthcare structure. This original strategy was retrospectively evaluated over a period of three years. The purpose of our study was not to assess the intrinsic performances of the methods, which were validated elsewhere. Instead, the aim of our work was to evaluate the practicability of a new process, its potential impact on patient management and the future challenges that needed to be addressed.

## Materials and Methods

### POC-lab facilities

**Figure 1:**
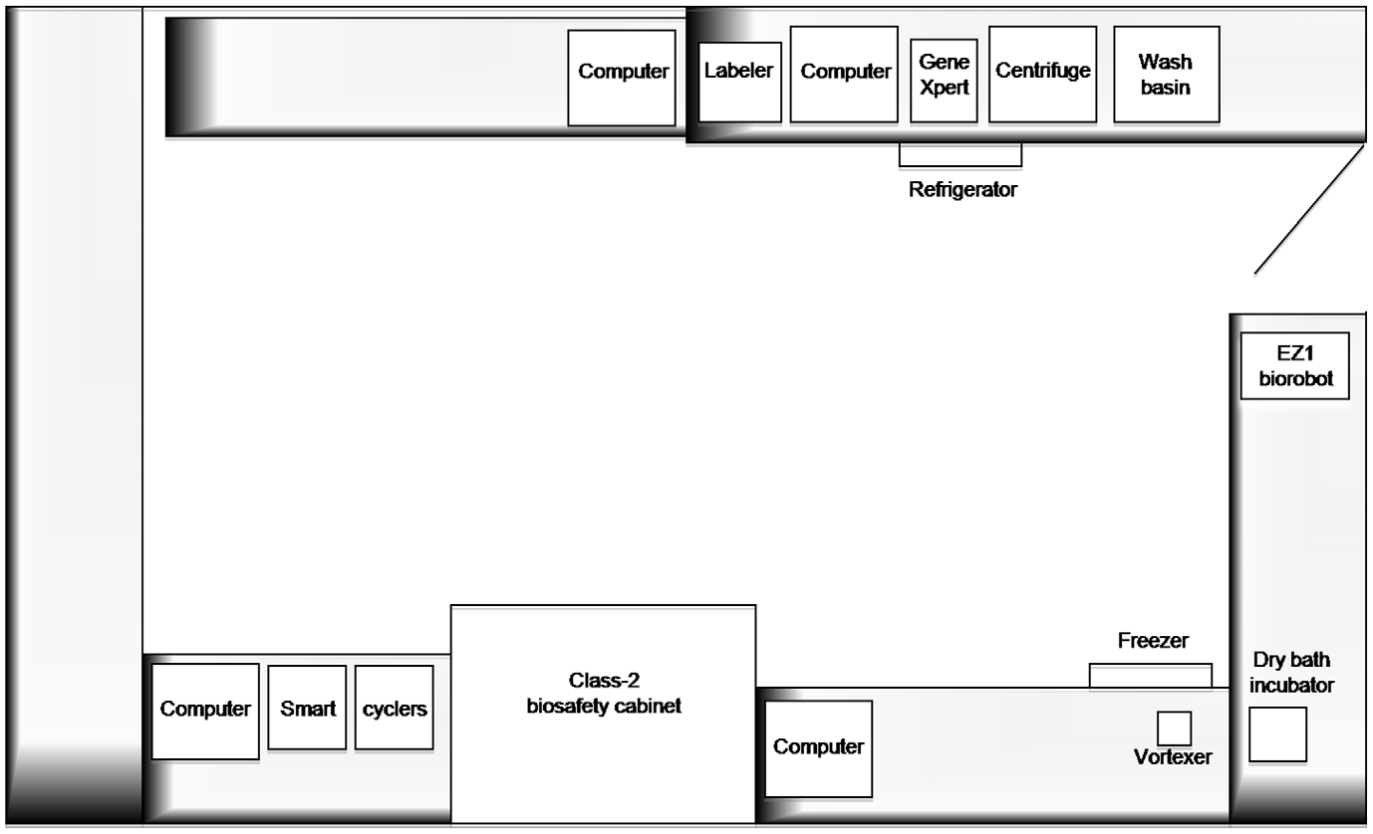
Layout of the POC-lab and equipment.

POC-labs were designed as autonomous structures running with minimal dedicated material and reagents, and they were contained in a single room. The sizes of the rooms were 18.8 m^2^ in la Timone and 18 m^2^ in Hôpital Nord (figure 1).

### POC-lab staff

Both POC-labs were organized to run 24 hours per day with a single operator, under the supervision of a medical microbiologist who was on call. The technical staff performing the tests was residents training in clinical microbiology in accordance with the French legislation (ordonnance n° 2010-49 du 13 janvier 2010 relative à la biologie médicale). Prior to working in the POC-labs, they were given a half-day teaching course provided by a medical microbiologist working in the core laboratory. The theoretical aspects of POC testing were reviewed, including nonconformities, principles of immuno-chromatographic tests (ICT), real-time PCR assays and the necessity for controls and interpretation of results. Five days of practical learning were required to learn all the quality procedures necessary for safe and appropriate testing. Written procedures illustrated with photos were also available on a intranet system to provide the technical staff with constant access to the instructions related to POC testing. A start menu gave access to the panel of POC testing, the locations where reagents were stored, detailed procedures for tests and pictures of test results.

One of the medical microbiologists working in the core laboratory was responsible for the medical validation of test results and could be reached at any time. The results were reviewed once daily in the morning. A weekly lab meeting was also prepared to display POC week activity and results, including the number of tests requested and the number and rate of positive results, using a Microsoft Excel table. The results of molecular tests were then compared on a weekly basis to the results of the tests performed in the core laboratory, such as culture, serology and additional molecular testing.

### POC-lab tests and procedures

The tests and procedures that were implemented in POC-labs were technically undemanding and included 13 immuno-chromatographic tests (ICTs), 2 agglutination assays and 3 commercially available and 5 laboratory-developed real-time PCR assays (table S1). The specimens were processed as soon as they were delivered, and the test turnaround time did not exceed 4 hours, varying between 30 minutes and 3.5 hours according to the tests.

The laboratory-developed PCR assays were first tested in the core laboratory before being implemented in the POC-labs. The choice of target genes relied on a literature review, and the design of primers and probes was performed using Primer3 software. The sequences of primers and probes are detailed in Table S2. The reference and clinical strains were used to set up the PCR procedures (details in text S1); the methods were then evaluated in clinical routine to validate the performances.

The ready-to-use mixes and amplification controls were respectively prepared in the reagent preparation clean room and the nucleic acid extraction room of the core laboratory and stored at −20°C. The positive and negative amplification controls were made with extracts from clinical specimens and nuclease-free distilled water, respectively. The validity of every newly-prepared batch of mixes and controls was checked; the positive controls were validated if the amplification signal was observed within an acceptable range (25–30 Ct). Consequently, the only steps the POC-lab staff was required to perform to run PCRs were the extraction step and the addition of fresh nucleic acid extracts. The results were defined as valid if 1) the negative control did not produce an amplification curve, and 2) the positive control produced an amplification signal within an acceptable range. Post-PCR manipulations were not performed in the POC-labs.

For the influenza tests, ICT was performed during the entire study period. During the 2009 pandemic, the detection of the A/H1N1 influenza virus was also performed by real-time RT-PCR in POC-labs, which occurred from June 23 until the end of August, as previously reported [15]. Afterwards, the burst of activity led to the removal of the H1N1 PCR assay from POC-labs. However, the extraction step was still performed in POC-labs, and the core laboratory could then provide a definitive result in less than 24 hours.

### Volume of POC demands and diagnoses

POC testing did not provide a substitute for the routine analyses performed in the core laboratory because tests were requested at the physicians' convenience. Distinct laboratory request forms were therefore created for POC and core lab testing. Each month, we quantified both the number of POC tests ordered by physicians as well as the test findings. The data were extracted using InfoCentre software and sorted by Microsoft Excel software. The complete list of patients admitted in the emergency department of Hôpital Nord in 2009 was extracted anonymously from the data bank ORUPACA.

### Outcomes resulting from POC testing

Four main outcomes were expected according to test results: isolation of contagious patients, avoidance of unnecessary hospitalization, avoidance of unnecessary treatment (prescription of antiinfectious treatment avoided and/or reduced length of antiinfectious treatment) and the focusing of anti-infective therapy. Using this categorization, the number of theoretical outcomes resulting from POC-lab testing was assessed by focusing on the Hôpital Nord POC-lab. In the case of tonsillitis, the avoidance of empiric amoxicillin treatment was assessed using a 70-kg adult as a reference (1 g twice daily for 6 days). In the case of enterovirus meningitis, the mean length of the hospital stay of infected patients during the study period from 2008 to 2010 was measured and compared with that of infected patients during the 2005 outbreak [16]. From 2008 to 2010, enterovirus was detected in POC-labs by using the GeneXpert system (Cepheid, Sunnyvale, CA), whereas in 2005, the diagnosis was performed in the core laboratory using a laboratory-developed real-time RT-PCR assay [16]. To evaluate the respective impact of POC testing on patient management for bacterial meningitis, the number of diagnoses provided by POC testing was compared with that provided by microscopic examination and culture. For meningitis, testing requests that were collected from both POC-labs were considered to obtain a more representative sample of patients.

### Statistics

All data were analyzed with EpiInfo software (version 3.5.1, CDC, USA). Continuous variables were compared using analysis of variance or, when the data were abnormally distributed, the Mann-Whitney/Wilcoxon two-sample test. Proportions were compared by using the table function of EpiInfo software. The Mantel Haenszel test was used except when an expected cell value was less than 5, so the Fisher exact test was used. Statistical significance was defined as a p value <0.05.

### Ethical aspects

Following national regulations under the term of Biomedical Research (Loi Huriet-Sérusclat), a patient's signature at the hospital entrance office authorizes all the samples taken during hospitalization for diagnostic purposes to be accessible for research without specific consent from the patient, excluding those for human genetic research. Thus, ethics approval was not requested according to the Loi Huriet-Sérusclat.

## Results

### Integration of POC-lab tests within the diagnosis process

A total of 51,179 test requests were collected, including 8,605, 26,055 and 16,519 requests in 2008, 2009 and 2010, respectively (table S3). The la Timone and Hôpital Nord POC-labs collected 57% and 43% of these tests, respectively. The highest demand came from the emergency wards, with a smaller demand from the pediatrics and infectious diseases departments. The emergency, pediatrics and infectious diseases requests accounted for 73%, 10%, and 9% of the Hôpital Nord POC-lab requests and 60%, 14% and 4% of the la Timone POC-lab requests, respectively. A total of 6,244 positive results were provided, with 12% of the tests that were performed resulting in a positive result.

**Figure 2:**
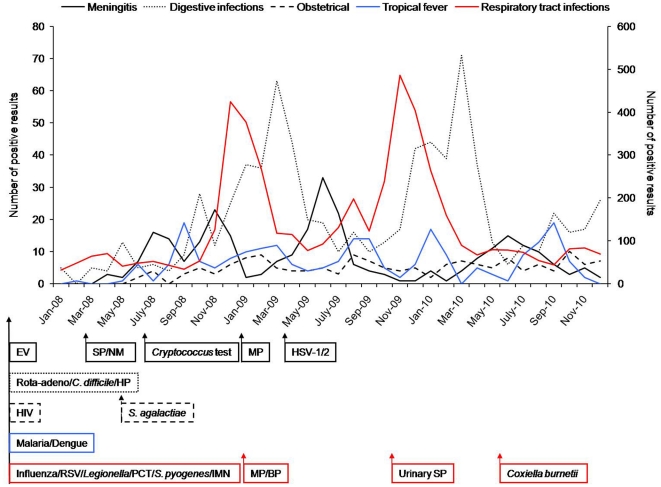
Kinetics of the diagnoses resulting from POC testing from 2008 to 2010. Left vertical axis: the number of positive results provided for meningitis, gastrointestinal infections, obstetric infections and tropical fever. Right vertical axis: the number of positive results provided for respiratory infections. Under the graph, the implementation of tests is indicated by arrows in chronological order. BP: *B. pertussis*; EV: enterovirus; HP: *H. pylori*; IMN: infectious mononucleosis; MP: *M. pneumoniae*; NM: *N. meningitidis*; PCT: procalcitonin, Rota/adeno: rotavirus/adenovirus; RSV: respiratory syncytial virus; SP: *S. pneumoniae*.

Testing related to respiratory tract infections (as listed in table S1) represented 78% of the overall demand. A burst of influenza demands related to the A/H1N1 outbreak occurred in 2009 with an early peak of diagnoses observed as early as August of 2009 (figure 2). From June 2009 through April of 2010, 1,075 tests out of 10,609 demands were positive for the influenza A virus, compared with 520 positive tests out of 3,491 demands from November of 2008 through April of 2009 (86% of A influenza virus). The diagnostic yields peaked at 15% in November 2009 in comparison to the previous influenza epidemic, which peaked at 25% in January of 2009. Alternatively, the number of respiratory syncytial virus diagnoses was stationary from 2008 to 2009 and from 2009 to 2010, regarding the period spreading from November through April (522 and 506 diagnoses, respectively).

The demand related to meningitis resulted in 4,097 tests. Enterovirus was the most frequently detected pathogen (234 diagnoses), with a peak of detection during summer and fall. A bacterial pathogen was detected in 49 patients, and herpes simplex virus-1/2 (HSV-1/2) was detected in six patients. For digestive infections, 4,380 tests were prescribed and 756 diagnoses were made, with rotavirus being the most frequently detected pathogen (613 diagnoses). Intrapartum detection of *S. agalactiae* in vaginal samples was positive in 16% of tested patients. With regard to HIV infection, 11 positive patients were diagnosed among 869 serologic tests, and 62% of those requests came from wards other than obstetrics because indications were extended to blood exposure accidents. In febrile patients returning from tropical countries, malaria was diagnosed in 223 patients out of 1,026 tests, and 85% of these 223 diagnoses were for *Plasmodium falciparum*.

### Impact of POC testing on patient management

**Figure 3:**
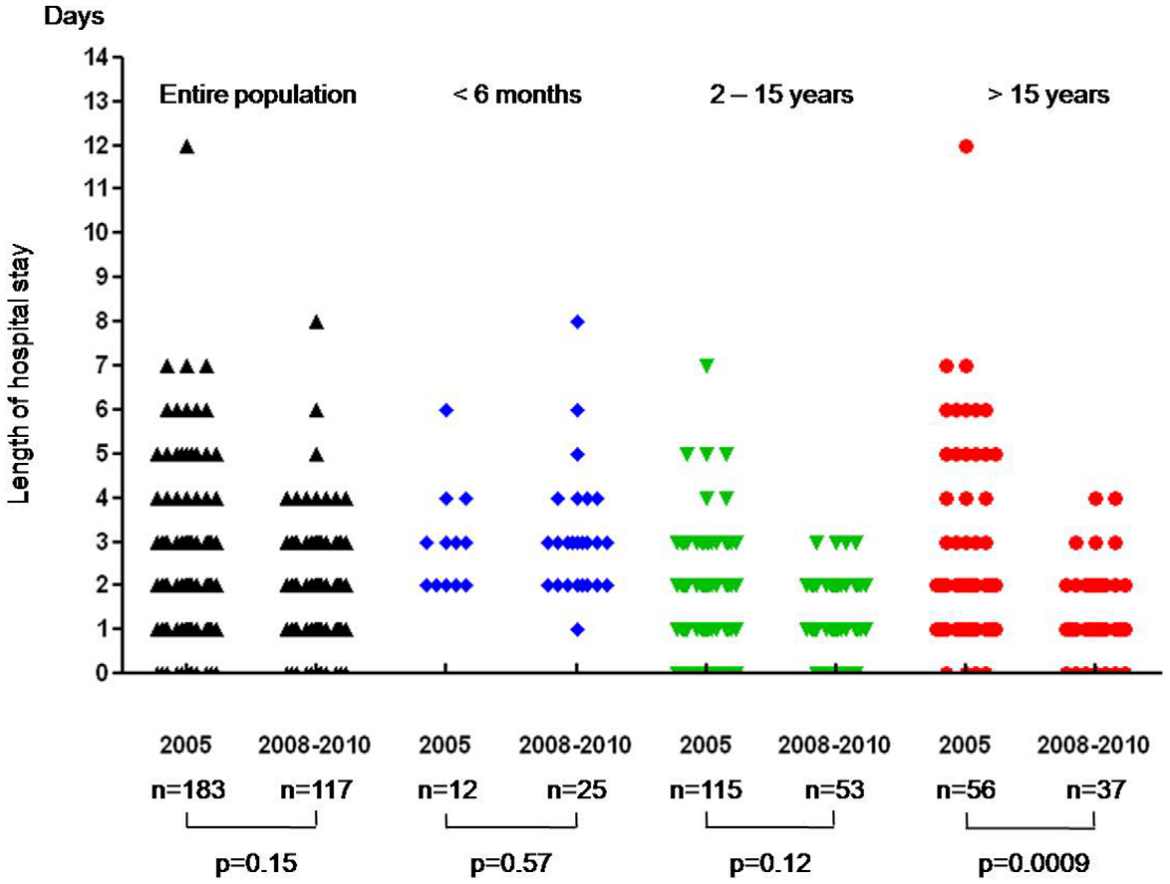
Differential length of the hospital stay of enterovirus-infected patients according to the diagnostic process. The molecular diagnosis of enterovirus meningitis was performed by the core laboratory in 2005 (laboratory developed real-time PCR assay) and the POC-labs from 2008 to 2010 (GeneXpert® system). The length of stay of infected patients in 2005 was measured [16] and compared with that of infected patients during the period from 2008 to 2010. Each symbol represents a patient with enterovirus meningitis. Statistical significance was defined as a p value <0.05.

The test results were subsequently sorted according to the expected outcomes of patients that presented to the emergency wards of the Hôpital Nord. The Hôpital Nord POC-lab detected a transmissible agent in 982 patients (table 1). The POC strategy was particularly crucial during the A/H1N1 influenza outbreak because only 11% of 3,097 tested patients were positive, and this avoided unnecessary isolation measures and treatment by oseltamivir in most cases. Similarly, treatment with amoxicillin might have been avoided for 1,844 of the 2,497 patients tested for tonsillitis, as well as for 763 of the 913 women of unknown status who were tested for *S. agalactiae*. A total of 11,064 days of empiric amoxicillin treatment might have been avoided in patients with negative *Streptococcus pyogenes* antigen tests and infectious mononucleosis diagnoses. In cases of bacterial meningitis, the microscopic examination and cerebrospinal fluid culture were negative in 25 of the 49 infected patients, so the POC tests were the only diagnostic tests used to guide antibiotic treatment. With regard to enterovirus meningitis, the mean length of the hospital stay decreased from 2.91±2.31 days in 2005 to 1.43±1.09 days from 2008 to 2010 (P = 0.0009) within the subgroup of infected patients over 15 years old (figure 3); no significant difference was highlighted within the other age groups or the entire population.

**Table 1:** POC test results according to expected outcomes.

Outcome	Test result	n*	Reference
Isolation for contagiousness	Positive influenza detection	545	[15], [17]
	(A/H1N1)	(335)	
	Positive RSV detection	320	[18]
	Positive *B. pertussis* detection	14	[19]
	Positive rotavirus/adenovirus detection	96	[20]
	Positive *C. difficile* detection	7	[21]
Avoid unnecessary hospitalization	Positive enterovirus detection	117	[9], [16]
Avoid unnecessary treatment	Positive RSV detection	320	[22]
	Negative procalcitonin detection	294	[23]
	Negative *S. pyogenes* detection	1,827	[24]
	Infectious mononucleosis diagnosis	17	[25]
	Positive enterovirus detection	117	[26]
	Negative *S. agalactiae* detection	763	[13]
	Dengue diagnosis	9	[27]
	*C. tetani* antibodies	8	[28]
Replace empiric with documented treatment	Positive A/H1N1 influenza detection	335	[15], [29]
	Presence of urinary pneumococcal antigens	10	[30]
	Presence of urinary *L. pneumophila* antigens	9	[31]
	Positive *M. pneumoniae* detection	21	[32]
	Bacterial meningitis	13	[33]
	HSV meningitis	1	[34]
	Malaria	149	[35]

*Only test requests coming from the emergency wards of the Hôpital Nord were considered. HSV: herpes simplex virus, RSV: respiratory syncitial virus.

Lastly, POC testing was performed in 8% of 66,810 patients admitted to the emergency wards of the Hôpital Nord in 2009. These patients were immediately discharged 2.6 times more often than patients tested with a conventional diagnostic procedure.

## Discussion

Our work is a retrospective observational study that provides preliminary data on the practical issues and challenges of a new organizational process implemented at the “hospital's doorstep”. POC-labs implemented inside emergency departments have been previously described by Lee-Lewandrowski et al. [36], but they studied the impact of chemical analyses on patient flow. In this study, our attention was primarily focused on the Hôpital Nord POC-lab, which corresponds to the previously described model of satellite laboratories [10], [36], [37]. It is important to stress that POC testing did not substitute for any routine analysis, and it is obvious that the experience was successful because a large demand developed afterward. It is also important to consider that the demand of laboratory tests is regulated by budgetary limits because chief physicians regularly inspect the benefits and expenses made by their department. The demands for POC testing would have certainly been reduced if unreasonable cost overrun had been highlighted.

Our evaluation was inherently limited regarding the impact of POC-labs on patient management. The actual value of care achieved with the implementation of POC-labs should be measured in a cost-effectiveness study, by assessing in a controlled manner the patient outcomes (measured by health recovery, sequelae and recurrences) relative to the costs (related to the length of hospital stay, additional testing, drug prescriptions and adverse effects) in a model comparing a conventional and a POC-lab driven diagnostic procedure.

However, a first comparative analysis was carried out between the POC-driven and the routine diagnosis of enterovirus meningitis. Enterovirus meningitis is a benign disease and does not warrant hospitalization; the exception is for infections related to the enterovirus 71 type, for which the prognosis may be different when it involves the central nervous system [38]. When the 2000 outbreak in Marseilles was reported, the mean length of the hospital stay of enterovirus-infected patients was 5.5±4.9 days [9]. Later, the implementation of PCR-based diagnosis as a routine method correlated with a significant decrease of mean length of hospitalization to 2.2±1.8 days during the 2005 outbreak [16]. The results from this study showed that mean length of the hospital stay was reduced by 1.5 days within the subgroup of infected patients over 15 years old. The fact that a significant reduction was not achieved within the pediatric population could be related to the lack of an observation unit in the pediatric emergency wards. Actually, a mean delay time of four hours was measured between the admission of children with enterovirus meningitis and the reception of lumbar puncture in POC-labs (data not shown). Therefore, the patients are already hospitalized before the POC result is available, while an observation of a few hours and symptomatic care should be conducted before these patients are home-discharged.

The POC-lab strategy modified the management of contagious diseases. Nosocomial transmission of contagious pathogens was previously illustrated by outbreaks of influenza virus [17], respiratory syncytial virus [18], *Bordetella pertussis* [19], rotavirus [20] and *Clostridium difficile* [21]. In these situations, it is advisable to consider every suspected patient as a potentially contagious source, which leads either to unnecessary procedures and costs or to the isolation of suspected patients until the delivery of test results. These results may be delayed, leading to patient transfer from one ward to another [17]. The POC-lab strategy allowed the appropriate procedures to be performed with only short delays in 982 patients who presented at the emergency wards of the Hôpital Nord. During the initial phase of the A/H1N1 outbreak, the PCR-based diagnosis implemented in June of 2009 allowed for rapid triage (4–7 hours) of patients presenting with influenza-like illness [15]. At the peak period, the burst of activity led to the removal of H1N1 molecular tests from POC-labs. However, the extraction step was still performed in POC-labs, and the core laboratory provided a definite result within 10 to 24 hours. Moreover, ICT could detect 57.7% of infected patients in less than two hours [15], [29], [39], [40]. The use of the POC-lab was therefore an optimal strategy to prevent contagiousness and to aid in focused treatment in outbreak situations.

Another issue was to prevent unjustified prescriptions. France has been identified as one of the nations with the highest antibiotic use, both in Europe and worldwide [41]. This concern was reinforced by the fact that practitioners could not use waived tests, such as the rapid detection of *S. pyogenes* in tonsillitis, because the French government does not approve reimbursement. In this study, we highlighted 3,347 opportunities to avoid unnecessary antibiotic treatment in the emergency wards of the Hôpital Nord, including opportunities involving viral infections. Specifically, these infections included respiratory syncytial virus, mononucleosis, enterovirus and dengue fever, along with negative *S. pyogenes* and *S. agalactiae* detection. A procalcitonin assay was implemented upon the request of emergency practitioners for the same purpose. This is because low serum concentrations make a bacterial infection unlikely in the context of community-acquired pneumonia and allow doctors to withhold antibiotic treatment without compromising patient care [42]. However, performing a quantitative assay validated procalcitonin-guided antibiotic use. This assay was able to detect lower values of procalcitonin than ICT [43], [44], particularly for values within the range of uncertainty (0.25–0.5 µg/L), in which case a bacterial infection is possible and antimicrobial treatment is advised [42]. Nevertheless, the quantitative assay was also shown to be easily practicable at the site of care [23].

Lastly, the POC-lab directed the choice of antimicrobial therapy. In half of the bacterial meningitis cases, microscopic examination of CSF and cultures were negative [33], partly due to the prescription of third-generation cephalosporins before the lumbar puncture was performed (data not shown). Consequently, only an empiric treatment combining a third-generation cephalosporin and vancomycin is followed, with vancomycin prescribed systematically in children [45] and recommended in adults when a pneumococcal resistance to β-lactam is possible [46]. Testing in POC-labs therefore might have avoided the prescription of vancomycin for meningococcal meningitis and in certain cases of pneumococcal meningitis. In the case of malaria, we previously reported that two-thirds of the infected patients in Marseilles were Comorian migrants and that *P. falciparum* was responsible for more than 90% of these cases [47]. When microscopic evaluation in the core laboratory is not available, rapid diagnostic tests are useful in this population because they usually harbor a high level of parasitemia [35].

We assume that clinical microbiology in the 21^st^ century will focus on concerns regarding the real-time management of patients by delivering results at the time of care. To our knowledge, this is the first report in the field of clinical microbiology of a strategy closely implemented into patient care. We could only provide preliminary data about its potential benefits, but we support the emerging concept that it is possible to make a diagnosis based essentially on a molecular or immunochromatographic approach that produces profound changes in patient care.

## Supporting Information

Table S1 **Primers and probes used for laboratory developed PCR assays.**
(DOC)

Table S2 **List of POC-lab tests.**
(DOC)

Table S3 Prescriptions and diagnoses of POC-lab tests from 2008 to 2010.
(DOC)

Text S1 **Methods used for POC testing.**
(DOC)
