# Supplementary material for: Revolutionizing Clinical Microbiology Laboratory Organization in Hospitals with In Situ Point-of-Care
Source: PLoS One. 2011 Jul 19;6(7):e22403. doi: 10.1371/journal.pone.0022403 (PMC3139639; doi:10.1371/journal.pone.0022403)
Supplement: Table S1 — Primers and probes used for laboratory developed PCR assays. (DOC) [file pone.0022403.s001.doc]

**Table S1**. List of POC-lab tests (adapted from Nougairède et al. [15]).

| Syndrome | Pathogen | Specimen | Test | Manufacturer | Turnaround time |
| --- | --- | --- | --- | --- | --- |
| Respiratory | Influenza virus | Naso-pharyngeal aspirate | ICT/Antigen | Becton Dickinson | 30 min |
|  | RSV | Naso-pharyngeal aspirate | ICT/Antigen | bioMérieux | 30 min |
|  | *S. pneumoniae* | Urine | ICT/Antigen | Inverness Medical | 30 min |
|  | *L. pneumophila* | Urine | ICT/Antigen | Inverness Medical | 30 min |
|  | *B. pertussis* | Sputum or naso-pharyngeal aspirate | Laboratory developed qPCR assay | - | 3.5 h |
|  | *M. pneumoniae* | Sputum or naso-pharyngeal aspirate | Laboratory developed qPCR assay | - | 3.5 h |
|  | *C. burnetii* | Sputum or naso-pharyngeal aspirate | Laboratory developed qPCR assay | - | 3.5 h |
|  | Procalcitonin | Serum | ICT/Antigen | BRAHMS | 45 min |
|  | *S. pyogenes* | Naso-pharyngeal swab | ICT/Antigen | Dectra Pharm | 30 min |
|  | EBV | Serum | Agglutination assay/Antibody | Oxoid | 30 min |
| Meningitis | Enterovirus | CSF | Commercially available qRT-PCR assay | Cepheid | 3 h |
|  | HSV-1/2 | CSF | Commercially available qPCR | Cepheid | 3.5 h |
|  | *S. pneumoniae* | CSF | Laboratory developed qPCR assay | - | 3.5 h |
|  | *N. meningitidis* | CSF | Laboratory developed qPCR assay | - | 3.5 h |
|  | *M. pneumoniae* | CSF | Laboratory developed qPCR assay | - | 3.5 h |
|  | *C. neoformans* | CSF | Agglutination assay/Antigen | Meridian Bioscience | 45 min |
| Digestive | Rotavirus-adenovirus | Stool/Rectal swab | ICT/Antigen | bioMérieux | 30 min |
|  | *C. difficile* | Stool/Rectal swab | ICT/Antigen | Techlab | 30 min |
|  | *H. pylori* | Stool/Rectal swab | ICT/Antigen | Meridian Bioscience | 30 min |
| Obstetrical | *S. agalactiae* | Vaginal swab | Commercially available qPCR | Cepheid | 2.5 h |
|  | HIV1/2 | Serum | ICT/Antibody | Inverness Medical | 30 min |
| Tropical | *P. falciparum* | Blood | ICT/Antigen | Core Diagnostics | 30 min |
|  | Dengue virus | Serum | ICT/Antigen and antibody | SD | 30 min |
| Other | *C. tetani* | Serum | ICT/Antibody | Zentech | 30 min |

CSF: cerebrospinal fluid, EBV: Epstein-Barr virus, HIV: human immunodeficiency virus, HSV: herpes simplex virus, ICT: immuno-chromatographic test, qPCR: quantitative real-time PCR, qRT-PCR: quantitative real-time reverse-transcriptase PCR, RSV: respiratory syncytial virus.
