# Supplementary material for: Revolutionizing Clinical Microbiology Laboratory Organization in Hospitals with In Situ Point-of-Care
Source: PLoS One. 2011 Jul 19;6(7):e22403. doi: 10.1371/journal.pone.0022403 (PMC3139639; doi:10.1371/journal.pone.0022403)
Supplement: Table S2 — List of POC-lab tests. (DOC) [file pone.0022403.s002.doc]

**Table S2. Primers and probes used for laboratory developed PCR assays**.

| Pathogen detected | Gene | Primers (5’-3’) | Probe | Tm |
| --- | --- | --- | --- | --- |
| *M. pneumoniae* | P1 gene | CCG TTT TAC TCG TGC CGC G | AGC GTG GTG TAC TAT GA | 60°C |
|  |  | GGG AGC GCT AAC CCC CG |  |  |
| *B. pertussis* | IS481 | CCG ACC TTA CCG CCC ACA G | CCA ATG GCA AGG CCG AAC GC | 60°C |
|  |  | ACG CAA GGC TGA CGT GA |  |  |
| *N. meningitidis* | *ctrA* | GCT GCG GTA GGT GGT TCA A | CAT TGC CAC GTG TCA GCT GCA CAT | 60°C |
|  |  | TTG TCG CGG ATT TGC AAC TA |  |  |
|  | *crgA* | GTT CAA GTG GTG GAA AGC GG | CAG TTG GCG ATG GCA | 60°C |
|  |  | TTT TCC TCC AGC CGT TTG AC |  |  |
| *S. pneumoniae* | *ply* | GCG ATA GCT TTC TTC AAG TGG | CCC AGC AAT TCA AGT GTT CGC CGA | 60°C |
|  |  | TA GCC AAC AAA TCG TTT ACC G | |  |
|  | *lytA* | CCT GTA GCC ATT TCG CCT GA | AGA CGG CAA CTG GTA CTG GTT CGA CAA | 60°C |
|  |  | GAC CGC TGG AGG AAG CAC A |  |  |
| *C. burnetii* | IS1111 | CAA GAA ACG TAT CGC TGT GGC | CCG AGT TCG AAA CAA TGA GGG CTG | 60°C |
|  |  | CAC AGA GCC ACC GTA TGA ATC | |  |
