# Supplementary material for: Revolutionizing Clinical Microbiology Laboratory Organization in Hospitals with In Situ Point-of-Care
Source: PLoS One. 2011 Jul 19;6(7):e22403. doi: 10.1371/journal.pone.0022403 (PMC3139639; doi:10.1371/journal.pone.0022403)
Supplement: Table S3 — Prescriptions and diagnoses of POC-lab tests from 2008 to 2010. (DOC) [file pone.0022403.s003.doc]

**Table S3**. Prescriptions and diagnoses of POC-lab tests from 2008 to 2010.

| Syndrome | Pathogen |  | 2008 |  |  | 2009 |  |  | 2010 |  |  | Total |  |
| --- | --- | --- | --- | --- | --- | --- | --- | --- | --- | --- | --- | --- | --- |
|  |  | Demands | Diagnoses | Diagnoses | Demands | Diagnoses | Diagnoses | Demands | Diagnoses | Diagnoses | Demands | Diagnoses | Diagnoses |
|  |  | (n) | (n) | (%) | (n) | (n) | (%) | (n) | (n) | (%) | (n) | (n) | (%) |
| Respiratory | influenza | 1390 | 102 | 7 | 12455 | 1489 | 12 | 2551 | 25 | 1 | 16396 | 1616 | 10 |
|  | RSV | 1537 | 378 | 25 | 3300 | 297 | 9 | 2171 | 451 | 21 | 7008 | 1126 | 16 |
|  | *S. pneumoniae* | - | - | - | 67 | 12 | 18 | 967 | 63 | 7 | 1034 | 75 | 7 |
|  | *L. pneumophila* | 168 | 1 | 1 | 405 | 4 | 1 | 954 | 9 | 1 | 1527 | 14 | 1 |
|  | *M. pneumoniae* | - | - | - | 668 | 25 | 4 | 1184 | 47 | 4 | 1852 | 72 | 4 |
|  | *C. burnetii* | - | - | - | - | - | - | 244 | 0 | 0 | 244 | 0 | 0 |
|  | *B. pertussis* | - | - | - | 561 | 20 | 4 | 761 | 10 | 1 | 1322 | 30 | 2 |
|  | Procalcitonin | 1169 | 206 | 18 | 1090 | 169 | 16 | 774 | 104 | 13 | 3033 | 479 | 16 |
|  | *S. pyogenes* | 1817 | 339 | 19 | 2912 | 567 | 19 | 2210 | 420 | 19 | 6939 | 1326 | 19 |
|  | EBV | 82 | 9 | 11 | 143 | 13 | 9 | 146 | 16 | 11 | 371 | 38 | 10 |
| Meningitis | EV | 296 | 84 | 28 | 482 | 92 | 19 | 416 | 58 | 14 | 1194 | 234 | 20 |
|  | HSV-1/2 | 3 | 0 | 0 | 114 | 3 | 3 | 251 | 3 | 1 | 368 | 6 | 2 |
|  | *S. pneumoniae* | 267 | 10 | 4 | 479 | 9 | 2 | 410 | 14 | 3 | 1156 | 33 | 3 |
|  | *N. meningitidis* | 272 | 6 | 2 | 480 | 3 | 1 | 403 | 6 | 1 | 1155 | 15 | 1 |
|  | *M. pneumoniae* | *-* | - | - | 38 | 1 | 3 | 144 | 0 | 0 | 182 | 1 | 1 |
|  | *C. neoformans* | 12 | 0 | 0 | 17 | 0 | 0 | 13 | 0 | 0 | 42 | 0 | 0 |
| Digestive | Rotavirus | 537 | 94 | 18 | 1075 | 273 | 25 | 1221 | 246 | 20 | 2833 | 613 | 22 |
|  | Adenovirus |  | 11 | 2 |  | 40 | 4 |  | 49 | 4 |  | 100 | 4 |
|  | *C. difficile* | 352 | 7 | 2 | 518 | 10 | 2 | 535 | 17 | 3 | 1405 | 34 | 2 |
|  | *H. pylori* | 33 | 4 | 12 | 49 | 4 | 8 | 60 | 1 | 2 | 142 | 9 | 6 |
| Obstetrical | *S. agalactiae* | 149 | 21 | 14 | 352 | 64 | 18 | 417 | 66 | 16 | 918 | 151 | 16 |
|  | HIV | 199 | 2 | 1 | 401 | 4 | 1 | 269 | 5 | 2 | 869 | 11 | 1 |
| Tropical | *Plasmodium* spp | 277 | 50 | 18 | 367 | 90 | 25 | 382 | 83 | 22 | 1026 | 223 | 22 |
|  | Dengue virus | 45 | 4 | 9 | 66 | 6 | 9 | 15 | 2 | 13 | 126 | 12 | 10 |
| Other | *C. tetani* | - | - | - | 16 | 8 | 50 | 21 | 18 | 86 | 37 | 26 | 70 |
| Total |  | 8605 | 1328 | 15 | 26055 | 3203 | 12 | 16519 | 1713 | 10 | 51179 | 6244 | 12 |

n: number of tests and diagnoses given in absolute values. %: diagnosis rate. RSV: respiratory syncytial virus, EBV: Epstein-Barr virus, EV: enterovirus, HSV: herpes simplex virus, HIV: human immunodeficiency virus.
