# Supplementary material for: Revolutionizing Clinical Microbiology Laboratory Organization in Hospitals with In Situ Point-of-Care
Source: PLoS One. 2011 Jul 19;6(7):e22403. doi: 10.1371/journal.pone.0022403 (PMC3139639; doi:10.1371/journal.pone.0022403)
Supplement: Text S1 — Methods used for POC testing. (DOC) [file pone.0022403.s004.doc]

**SUPPORTING INFORMATION**

**Text S1. Methods used for POC testing.**

**Quantitative real-time PCR (qPCR)**

The automated GeneXpert Dx system (Cepheid) was used to diagnose enterovirus (Xpert EV, Cepheid, Sunnyvale, CA) and *Streptococcus agalactiae* (Xpert GBS, Cepheid) according to the manufacturer’s recommendations. To detect other pathogens, nucleic acids were extracted using the Biorobot EZ1 workstation (QIAGEN, Les Ulis, France), and PCR assays (final volume 25 µL) were performed using a two-block Smartcycler system (Cepheid). The detection of A/H1N1 influenza virus by real-time RT-PCR was performed in 2009 from June 23 until the end of August as previously described [15]. For herpes simplex virus-1/2 (HSV-1/2) detection, DNA extraction was performed using the EZ1 virus Mini Kit (QIAGEN) with a sample volume of 200 µL and buffer AVE volume of 60 µL (elution volume 90 µL). The reaction mixture was obtained by dissolving one bead of master mix (SmartMix HM, Cepheid) and one bead of probe and primers set (Smartcycler HSV1/2 reagents, Cepheid) in 40 µL of sterile water in a 1.5-mL tube. Five microliters of extracted DNA (or sterile water for negative controls) was then added to 20 µL of this reaction mixture. The reaction conditions were as follows: 95°C for 120 s, followed by 50 cycles of 95°C for 15 s, 57°C for 15 s and 72°C for 15 s. The detection of *Bordetella pertussis*, *Mycoplasma pneumoniae* in respiratory specimens and *Streptococcus pneumoniae*, *Neisseria meningitidis* and *M. pneumoniae* in CSF was performed using laboratory developed real-time qPCR assay. DNA was purified using the EZ1 DNA tissue kit (QIAGEN) with a modified protocol. One hundred microliters of clinical samples (sputum, naso-pharyngeal aspirate or CSF) was mixed with 138 µL of buffer G2 and 12 µL of proteinase K in a 2-mL cryotube, then heated at 70°C for 10 min before automated purification (elution volumes of 50 µL and 100 µL for respiratory specimens and CSF, respectively). Five microliters of pure or 1/10 diluted DNA was added to 20 µL of a reaction mixture containing 0.5 µL of probe **(**75 nM for *ply*, *lytA*, *ctrA*, and *crgA*; 100 nM for the P1 gene; 60 nM for IS481**)**, 0.5 µL of each primer **(**400 nM**)**, 0.5 U of uracil-N-glycosylase (Invitrogen, Cergy Pontoise, France), 12.5 µL of master mix (QuantiTect Probe PCR kit, QIAGEN) and sterile water. The reaction conditions were as follows: 50°C for 120 s, 95°C for 900 s, followed by 50 cycles of 95°C for 5 s and 60°C for 40 s.

**Immuno-chromatographic assays (ICTs)**

ICTs were used for the rapid detection of *Streptococcus pyogenes* (Streptatest, Dectra Pharm, Strasbourg, France), *Legionella pneumophila* serotype 1 (BinaxNOW Legionella, Inverness medical, Scarborough, ME), *S. pneumoniae* (BinaxNOW S. pneumoniae, Inverness medical), influenza virus A/B (Directigen EZ Flu A+B, Becton, Dickinson and Company, Sparks, MD), respiratory syncytial virus (Quickvue RSV, bioMérieux, Marcy l’Etoile, France), toxigenic *Clostridium difficile* (Tox A/B Quik ChekTM, Techlab Inc., Blacksburg, VA), rotavirus and adenovirus (VIKIA Rota-Adeno, bioMérieux, Marcy l’Etoile, France), *Helicobacter pylori* (ImmunoCard STAT! HpSA, Meridian Bioscience Inc., Cincinnati, OH), *Plasmodium* spp. (Core Malaria Pan/Pv/Pf, Core Diagnostics, Birmingham, UK), *Cryptococcus neoformans* (CALAS, Meridian Bioscience Inc.), procalcitonin antigens (PCT-Q, BRAHMS Diagnostica, GmbH, Berlin, Germany), antibodies to HIV-1/2 (Determine HIV-1/2, Inverness Medical Co., Chiba, Japan), Dengue virus (SD Bioline Dengue Duo, SD, Gurgaon, India), infectious mononucleosis heterophile antibodies (Infectious Mononucleosis Kit Oxoid, Dartford Kent, UK) and tetanus antigens and antibodies (Tetanos Quick Stick, Zentech, Liège, Belgium), according to the manufacturers’ recommendations.
